# Comprehensive monitoring of a special mixture of prominent endocrine disrupting chemicals in human urine using a carefully adjusted hydrolysis of conjugates

**DOI:** 10.1007/s00216-022-04438-0

**Published:** 2022-11-26

**Authors:** Heike Denghel, Thomas Göen

**Affiliations:** Institute and Outpatient Clinic of Occupational, Social and Environmental Medicine, Friedrich-Alexander-Universität Erlangen-Nürnberg, Henkestraße 9-11, 91054 Erlangen, Germany

**Keywords:** Phenols, Endocrine disrupting chemicals, Human biomonitoring, Enzymatic hydrolysis, Conjugates, GC-MS/MS

## Abstract

Many xenobiotics were identified as possible endocrine disruptors during the last decades. Structural analogy of these substances to natural hormones may lead to agonists or antagonists of hormone receptors. For a comprehensive human biomonitoring of such substances, we developed a simple, reliable, and highly sensitive method for the simultaneous monitoring of the parameters bisphenol A, triclosan, methylparaben, ethylparaben, propylparaben, butylparaben, benzophenone-1, benzophenone-3, 3,5,6-trichloropyridin-2-ol, *p*-nitrophenol, genistein, and daidzein in urine. Thereby, optimization of the enzymatic hydrolysis and the use of β-glucuronidase from *E. coli* K12 as well as sulfatase from *Aerobacter aerogenes* ensures the acquisition of intact analytes without cleavage of ester bonds among parabens. Validation of the method revealed limits of detection between 0.02 and 0.25 µg/L as well as limits of quantification between 0.08 and 0.83 µg/L. Thereby, the use of analyte-free surrogate matrix for calibration and control material influenced the sensitivity of the procedure positively. Furthermore, excellent precision in and between series was observed. Good absolute and relative recoveries additionally proved the robustness of the multimethod. Thus, the procedure can be applied for exploring the exposome to these prominent endocrine disruptors in the general population.

**Graphical Abstract:**

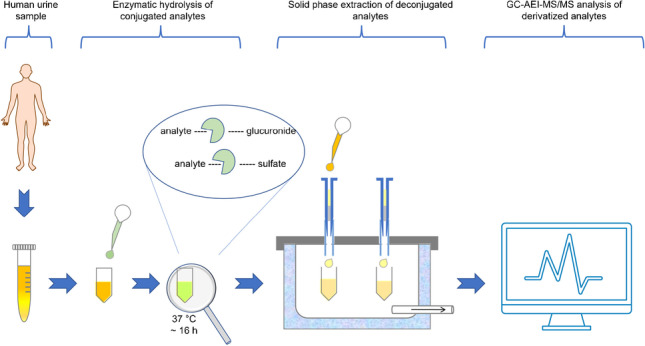

**Supplementary information:**

The online version contains supplementary material available at 10.1007/s00216-022-04438-0.

## Introduction

During the last decades, many xenobiotics which modulate the human endocrine system by different mechanisms were identified [1, 2]. A large group of these endocrine disrupting chemicals (EDC) features structural analogy to natural hormones, e.g., estradiol, and may act as agonists or antagonist of hormone receptors, e.g., estrogen receptors (ERs) [3]. Consequently, structurally similar substances like genistein and daidzein as well as further molecules with phenolic structures may interact with these receptors and thus belong to the described group of EDC [4].

One of the most prominent representative of these substances is bisphenol A (2,2-bis(4-hydroxy-phenyl)propane, BPA), for which effects on and through the endocrine system were demonstrated in vitro as well as in vivo [5–7]. BPA is applied as a key component to produce epoxy resins and polycarbonate plastics. An exposure of the population exists via the environment caused from emissions of chemical processes using BPA but also directly from consumer products [7]. Further phenolic substances of EDC potential, whose use in consumer products implicates a significant exposure of the population, are triclosan (TCS) and several esters of parahydroxybenzoic acid, so called parabens, which are frequently used as biocides in cosmetics and personal care products [8–11]. Other prominent phenolic substances with a comparable exposure pathway are benzophenones, which are used as UV filters and thus are ingredients of sunscreens but also cosmetics [12, 13]. A human exposure to relevant phenolic substances can also result from the degradation or metabolism of xenobiotics. Of particular interest are metabolites from frequently used pesticides which are carrying a phenolic moiety [14, 15]. Chlorpyrifos and chlorpyrifos-methyl are organophosphate pesticides, for which endocrine effects were demonstrated [16]. Both pesticides were degraded to 3,5,6-trichloropyridin-2-ol (2,3,5-trichloro-6-hydroxypyridine, TCPy) [17, 18]. Furthermore, TCPy can also derive from the metabolism of the herbicide triclopyr [19]. Another prominent pesticide product is para-nitrophenol (PNP), which is well known as metabolite of parathion [20]. However, the substance can also result from degradation and metabolism of parathion and *O*-ethyl *O*-(4-nitrophenyl)phenylphosphonothioate (EPN) [14].

Besides xenobiotics, the exposure to naturally occurring endocrine disruptors should be taken into account for a holistic exploration of the EDC exposure [21, 22]. Relevant in this approach are the phytoestrogens genistein (5,7-dihydroxy-3-(4-hydroxyphenyl)-4H-1-benzopyran-4-one, GEN) and daidzein (7-hydroxy-3-(4-hydroxy phenyl)-4H-1-benzopyran-4-one, DAI), which are consumed in considerable amounts via nutrition occasionally [23]. After absorption in the human body, GEN and DAI are eliminated via urine in conjugate forms mainly [24]. GEN and DAI exhibit strong ER binding and showed several adverse impact on human fertility and reproductive parameters, such as sperm motility and morphology [25–28].

In epidemiological studies, the questions, whether adverse effects are resulted from synergistic or additive effects of several compounds were addresse [29, 30]. The application of a multicomponent method for the simultaneous determination of a broad range of EDC biomarkers gives a crucial advantage to achieve these objectives. Several biomonitoring studies demonstrated a relevant exposure of the general population to bisphenol A, triclosan, parabens, benzophenone UV filters, phenolic pesticide metabolites, and phytoestrogens [23, 31, 32], which supports considering these compounds for an EDC relevant exposome. Nevertheless, the integrative approach for the human biomonitoring of these previously mentioned phenolic agents must consider several requirements and challenges. First, all phenolic compounds are excreted effectively via urine, however, after conjugation to glucuronic acid and sulfate [15, 31, 33, 34]. For determining the total exposure to the agent, an effective hydrolysis of these conjugates must be included in the procedures. However, the inclusion of parameters with ester groups, e.g., parabens, challenges this operation due to their degradation sensitivity and demands a careful adjustment of this analytical step.

**Fig. 1:**
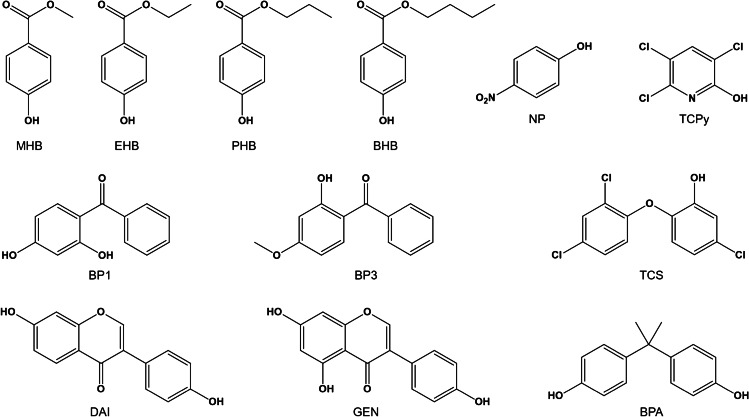
Chemical structures of the investigated analytes benzophenone-1 (BP1), benzophenone-3 (BP3), bisphenol A (BPA), n-butyl-4-hydroxybenzoate (BHB), daidzein (DAI), ethyl-4-hydroxybenzoate (EHB), genistein (GEN), methyl-4-hydroxybenzoate (MHB), n-propyl-4-hydroxybenzoate (PHB), 4-nitrophenol (NP), triclosan (TCS), and 3,5,6-trichloropyridinol (TCPy).

We developed a simple, reliable, and highly sensitive method for the simultaneous monitoring of the parameters BPA, TCS, MHB, EHB, PHB, BHB, BP1, BP3, TCPy, PNP, GEN, and DAI (Fig. 1) in human urine using gas chromatography coupled with tandem mass spectrometry (GC-MS/MS) considering the above mentioned requirements and challenges.

## Experimental

### Chemicals and materials

Benzophenone-1 (2,4-dihydroxybenzophenone, BP1), benzophenone-3 (2-hydroxy-4-methoxybenzophenone, BP3), bisphenol A (2,2-bis(4-hydroxyphenyl)propane, BPA), n-butyl-4-hydroxybenzoate (butylparaben, BHB), daidzein (7-hydroxy-3-(4-hydroxyphenyl)-4H-1-benzo pyran-4-one, DAI), ethyl-4-hydroxybenzoate (ethylparaben, EHB), genistein (5,7-dihydroxy-3-(4-hydroxyphenyl)-4H-1-benzopyran-4-one, GEN), methyl-4-hydroxybenzoate (methylparaben, MHB), n-propyl-4-hydroxybenzoate (propylparaben, PHB), 4-nitrophenol (NP), and triclosan (5-chloro-(2,4-dichlorophenoxy)phenol, TCS) were purchased from Sigma-Aldrich (Steinheim, Germany). 3,5,6-Trichloropyridin-2-ol (2,3,5-trichloro-6-hydroxypyridine, TCPy) was obtained from Chem Service (West Chester, USA). Figure 1 shows the chemical structures of all investigated analytes.

Isotope-labeled internal standards were included for every compound in terms of quantitative evaluations. Thereby, benzophenone-1-D5 (2′,3′,4′,5′,6′-^2^H_5_-2,4-dihydroxybenzophenone, BP1-D5, chemical purity 99 %, isotopic purity 99 %), 2,3,5,6-^2^H_4_-ethyl-4-hydroxybenzoate (ethylparaben-D4, EHB-D4, chemical purity 98 %, isotopic purity 98 %), 2,3,5,6-^2^H_4_-*n*-propyl-4-hydroxybenzoate (propylparaben-D4, PHB-D4, chemical purity 99 %, isotopic purity 99 %), and 2,3,5,6-^2^H_4_-*n*-butyl-4-hydroxybenzoate (butylparaben-D4, BHB-D4, chemical purity 98 %, isotopic purity 98 %) were delivered by Toronto Research Chemicals (Toronto, Canada). Benzophenone-3-D5 (2′,3′,4′,5′,6′-^2^H_5_-2-hydroxy-4-methoxybenzophenone, BP3-D5, chemical purity 99 %, isotopic purity 99 %), 2,2′,6,6′-^2^H_4_-bisphenol A (BPA-D4, chemical purity 99 %, isotopic purity 99 %), 2,3,5,6-^2^H_4_-methyl-4-hydroxybenzoate (methylparaben-D4, MHB-D4, chemical purity 99 %, isotopic purity 99 %), and triclosan-D3 (5-chloro-(3,5,6-^2^H_3_-2,4-dichlorophenoxy)phenol, TCS-D3, chemical purity 98 %, isotopic purity 98 %) were obtained from CDN isotopes (Quebec, Canada). 3′,5′,8-^2^H_3_-Daidzein (DAI-D3, 60 µg/mL in ACN, chemical purity > 98 %, isotopic purity 97 %) and 3′,5′,6,8-^2^H_4_-genistein (GEN-D4, 100 µg/mL in ACN, chemical purity > 98 %, isotopic purity 95 %) were purchased from Cambridge Isotope Laboratories (Andova, USA). 2,3,5,6-^2^H_4_-4-nitrophenol (NP-D4, chemical purity 98 %, isotopic purity 98 %) was delivered by Sigma Aldrich (Steinheim, Germany). 2,3,4-^13^C_3_-2,3,5-trichloro-6-hydroxypyridine (TCPy-^13^C_3_, chemical purity 99 %, isotopic purity > 95 %) was custom synthesized by the Institute for Organic and Biomolecular Chemistry (Göttingen, Germany).

*N*-*tert*-butyldimethylsilyl-*N*-methyltrifluoroacetamide (MtBSTFA) was acquired for derivatization from Sigma-Aldrich (Steinheim, Germany). For hydrolysis, β-glucuronidase from *E. coli K12* and β-glucuronidase/arylsulfatase from *Helix pomatia* were obtained from Roche Diagnostics GmbH (Mannheim, Germany). Sulfatase from *Aerobacter Aerogenes* and β-glucuronidase from *Helix pomatia* Type H-1 (partially purified powder, ≥300,000 units/g solid) were delivered by Sigma-Aldrich (Steinheim, Germany). Acetonitrile (ACN, dry, GC grade), acetic acid (glacial), methanol (MeOH, dry, GC grade) and toluene (dry, GC grade) as well as ammonium acetate (NH_4_Ac, for analysis), sodium acetate (NaAc, for analysis), and sodium chloride (NaCl, for analysis) were purchased from Merck KgaA (Darmstadt, Germany). Double distilled water was generated using a milli-Q-system (Millipore, Bedford, USA). Polystyrene divinylbenzene copolymer columns for solid-phase extraction (SPE) (Isolute® 101, 100 mg sorbent, 1 mL capacity, average particle size 65 µm (irregular-shaped particles), pore diameter 100 Å) were supplied from Biotage AB (Uppsala, Sweden). Artificial urine medium was purchased from Pickering Laboratories, Inc. (Mountain View, USA).

### Preparation of standards and hydrolysis buffer

Stock solutions of BP1, BP3, MHB, EHB, PHB, BHB, NP, TCPy, and TCS were set up by dissolving 1000 mg/L of the reference substances in ACN. In contrast, stock solutions of BPA, DAI, and GEN were prepared in EtOH in a concentration of 1000 mg/L. By dilution of the stock solutions with ACN, an analyte working mix containing 1.5 mg/L DAI and GEN as well as 0.5 mg/L of the remaining analytes was prepared. Stock solutions of the internal standards were set up in ACN by dissolving the reference substances BP1-D5, BP3-D5, BPA-D4, MHB-D4, EHB-D4, PHB-D4, BHB-D4, NP-D4, TCPy-^13^C_3_, and TCS-D3 in concentrations of 1000 mg/L each. Stock solutions of DAI-D3 and GEN-D4 were received by the supplier in concentrations of 60 µg/mL in ACN-D3 and 100 µg/mL in ACN, respectively. By dilution of the stock solutions with double-distilled water, an internal standard mix was prepared containing 1.5 mg/L GEN and DAI as well as 500 µg/L of the remaining analytes. For the hydrolysis buffer, 19.5 g NH_4_Ac were weighed in a 250-mL volumetric flask filled up with double-distilled water. The pH value was adjusted to a value of 6.5 through the addition of glacial acetic acid. The buffer was stored in the refrigerator at 4 to 8 °C and prepared freshly every 4 weeks.

### Calibration procedure

Seven concentration levels were applied for calibration. Therefore, 0.9 % NaCl (w/v) was spiked with different volumes of the analyte working mix to achieve final concentrations of 0.5, 1, 2, 5, 10, 25, and 50 µg/L of BP1, BP3, BPA, MHB, EHB, PHB, BHB, NP, TCPy, and TCS. In contrast, seven calibration levels containing 1.5, 3, 6, 15, 30, 75, and 150 µg/L of DAI and GEN were prepared. The calibration samples were stored frozen as aliquots of 1 mL at −20°C until analysis. Additionally, a blank sample consisting of 0.9 % NaCl (w/v) was integrated in every set of samples. For analysis, the calibration standards were treated according to the standard procedure in the “Sample preparation (final procedure)” section. Finally, the ratios of the analytes’ peak areas to the peak areas of the respective internal standards were illustrated as function of spiked concentration to obtain linear calibration curves. In terms of error correction, a specific isotopically labeled reference substance was included as internal standard for every analyte of interest.

### Sample preparation (final procedure)

Before analysis, the used glassware was heated to 150°C over night. Furthermore, disposable nitrile gloves were worn during laboratory work. Initially, frozen urine samples were thawed, equilibrated to room temperature, and mixed by vortexing. Afterwards, a hydrolysis step was performed to cleave possible glucuronide or sulfate conjugates. Therefore, 20 µL of the internal standard working solution and 500 µL of 1 M NH_4_Ac hydrolysis buffer (pH 6.5) were added to aliquots of 1 mL urine in 1.8 mL screw glass vials. Afterwards, 10 µL of β-glucuronidase K12 from *E. coli* and 10 µL of sulfatase from *Aerobacter aerogenes* were supplemented, and the samples were incubated over night at 37 °C. For solid phase extraction, Isolute® 101 cartridges were preconditioned successively with 500 µL MeOH, 250 µL ACN and twice with 500 µL of 1 M NH_4_Ac hydrolysis buffer (pH 6.5) and loaded with the hydrolyzed urine samples. A washing step with 500 µL buffer, 750 µL double-distilled water and 500 µL 50% MeOH was included. Afterwards, the SPE cartridges were centrifuged for 10 min at 1900 × g and dried for another 10 min under vacuum. The samples were eluted with 4 × 200 µL of ACN into 1.8 mL screw glass vials containing 200 µL of toluene as a keeper. The eluates were concentrated to a volume of 100 µL using a gentle stream of nitrogen. Afterwards, derivatization was performed (see the “Derivatization process (final procedure)” section).

To assess possible sample contamination with ubiquitously free BPA or with DAI and GEN from contaminated enzyme solution [35], every sample preparation series contained a blank sample, consisting of all used reagents and purified water instead of urine.

### Derivatization process (final procedure)

Derivatization was performed by the addition of 30 µL MtBSTFA to the concentrated sample extracts. After gentle vortexing and a reaction time of 10 min at room temperature, the samples were transferred to micro vials and measured via GC-AEI-MS/MS (see the “Gas chromatography- tandem mass spectrometry (GC-MS/MS)” section).

### Exploration of the sample preparation conditions

Sample preparation and derivatization were adjusted to optimize the determination of all parameters. Furthermore, surrogate matrices for calibration and control materials were tested as an alternative to pooled human urine. The conditions for the enzymatic hydrolysis of conjugates with glucuronic acid or sulfate were optimized.

#### Applicability of the derivatization procedure

The suitability of MtBSTFA as derivatization agent was verified for the defined analytical spectrum. Therefore, 100 µL of solutions containing 1 mg/L of BP1, BP3, MHB, EHB, PHB, BHB, NP, TCPy, and TCS in toluene were silylated with 30 µL of MtBSTFA according to the standard procedure in the “Derivatization process (final procedure)” section and analyzed in full-scan mode. For DAI and GEN, stock solutions of 200 mg/L were directly derivatized with 30 µL of MtBSTFA according to the final procedure without further dilution. An analysis blank containing pure toluene without any analyte was prepared and measured analogously to identify the relevant analyte signals and determine their retention times for the applied temperature program (see the “Gas chromatography- tandem mass spectrometry (GC-MS/MS)” section).

#### Suitability of matrices for calibration and control material

The suitability of human urine and surrogate matrices was investigated for the preparation of calibration and quality control material. As the use of analyte-free surrogates instead of contaminated pooled human urine samples was already successfully implemented in previous studies [36, 37], different matrices were tested, among them pooled human urine and two surrogates including 0.9% sodium chloride solution and artificial urine.

To this end, three-point calibration curves containing 2, 10, and 50 µg/L BP1, BP3, BPA, MHB, EHB, PHB, BHB, NP, TCPy, and TCS as well as 6, 30, and 150 µg/L DAI and GEN were spiked and analyzed in triple in pooled human urine, 0.9% sodium chloride solution, and artificial urine, respectively. The resulting averaged calibration curves in the different matrices were compared for all investigated analytes. Thereby, the slope of the averaged calibration curve in human urine was used as a reference equally to 100%. Moreover, the slopes of the averaged calibration curves in sodium chloride solution and artificial urine were related to this reference point. Values between 80 and 120% were assumed acceptable.

#### Optimization of the enzymatic hydrolysis

In a first approach, four different modifications of the enzymatic hydrolysis step were tested. Thereby, deconjugation with 10 µL β-glucuronidase/arylsulfatase from *Helix pomatia pH 5* (A) was compared with three further incubations: β-glucuronidase Type H-1 from *Helix pomatia pH 5* (B), 10 µL β-glucuronidase from *E. coli K 12* pH 5 (C), and 10 µL β-glucuronidase from *E. coli K 12* together with 10 µL sulfatase from *Aerobacter aerogenes Type VI pH 5* (D). Thereby, 1 mL aliquots of human urine samples containing glucuronide and sulfate conjugates of the investigated analytes were prepared with 500 µL of 0.4 M NaAc buffer (pH 5) (procedures (A), (C), and (D)) and 20 µL of the internal standard mix in 1.8 mL screw vials. Afterwards, hydrolysis enzymes were added according to the respective approach. In case of procedure (B), 40 mg of β-glucuronidase Type H-1 from *Helix pomatia* were weighed into 10 mL 0.4 M NaAc buffer (pH 5). Afterwards, 500 µL of this mixture and 20 µL of the internal standard mix were applied to 1 mL urine. The mixtures were incubated at 37°C over night and further processed according to the final operating procedure (see the “Sample preparation (final procedure)” subsection) using 0.4 M NaAc buffer (pH 5) instead of 1 M NH_4_Ac buffer (pH 6.5).

In a second approach, another four different modifications were tested for the enzymatic hydrolysis. Again, deconjugation with 10 µL β-glucuronidase/arylsulfatase from *Helix pomatia pH 5* (A) was used as a reference. Furthermore, 10 µL β-glucuronidase from *E. coli K 12* together with 10 µL sulfatase from *Aerobacter aerogenes Type VI* were tested for pH 5 (B) and 6.5 (C). Additionally, 10 µL β-glucuronidase from *E. coli K 12* together with 50 µL sulfatase from *Aerobacter aerogenes Type VI* were prepared at pH 6.5 (D). Thereby, 1 mL aliquots of human urine samples containing glucuronide and sulfate conjugates of the investigated analytes were prepared with 500 µL hydrolysis buffer (1 M NH4Ac buffer pH 6.5 or 0.4 M NaAc buffer pH 5, respectively) and 20 µL of the internal standard mix in 1.8 mL screw vials. Afterwards, hydrolysis enzymes were added according to the respective approach. Again, the mixtures were incubated at 37°C over night and further processed according to the final operating procedure (see “Sample preparation (final procedure)” subsection).

### Gas chromatography-tandem mass spectrometry (GC-MS/MS)

For GC-MS/MS, a Thermo Scientific TRACE 1310 gas chromatograph equipped with a split/splitless injector with a deactivated single taper helix liner, a Thermo Scientific TriPlus RSH autosampler, and a Thermo Scientific TSQ 9000 triple quadrupole mass spectrometer with an advanced electron ionization (AEI) source installed (Thermo Fisher Scientific Waltham, USA) were used. Chromeleon Software Version 7.2.8 was applied for device control and data analysis. A volume of 1 μL sample was injected into the system in splitless mode with a purge flow of 3.0 mL/min to vent after 1.2 min. The injector temperature was set to 320°C. A 5%-phenylarylene/95%-dimethyl polysiloxane low-bleed capillary column (ZB5 ms plus, 30 m × 250 μm × 0.25 μm) (Phenomenex, Aschaffenburg, Germany) was used for gas chromatographic separation at a constant flow of 1.2 mL/min helium carrier gas. The oven temperature was initially held at 120°C for 2 min. Afterwards, the temperature was raised in two steps: the first ramp was heated with a rate of 20°C/min up to 210°C and finally increased with 65°C/ min to 320°C. The final temperature was held for 9 min. A total run time of 17.2 min resulted for the chromatographic run. The transferline was held at 280°C. The ion source was operating at a temperature of 250°C with an electron energy of 50 eV in timed acquisition mode. Argon was used as collision gas. The mass spectrometer was working in multiple reaction monitoring (MRM) mode to detect the eluting analytes. The MS/MS operating conditions were adjusted via Auto SRM using a derivatized mixture of all investigated analytes (see the “Applicability of the derivatization procedure” subsection). Thereby, mass transitions of the most sensitive and selective precursor ions were optimized concerning product ions and corresponding collision energies (CE). Mass transitions with higher sensitivities were chosen as quantifiers for quantification, while two others were applied as qualifiers to further verify the compounds of interest.

### Quality control

Quality control material was prepared by spiking analyte free matrix (0.9 % NaCl (w/v)) with the investigated analytes in two different concentration levels. Thereby, Q _low_ was spiked with 1 µg/L of BP1, BP3, BPA, MHB, EHB, PHB, BHB, NP, TCPy, and TCS as well as 10 µg/L of GEN and DAI. In contrast, Q _high_ contained 10 µg/L of BP1, BP3, BPA, MHB, EHB, PHB, BHB, NP, TCPy, and TCS as well as 100 µg/L of GEN and DAI. Aliquots of 1 mL of the control material were stored frozen at −18°C until analysis. To every analysis series, one sample of Q _low_ and Q _high_ was included.

### Validation

Limits of detection (LOD) and quantification (LOQ) were calculated according to the calibration method as described in guideline DIN 32 645 [38]. Therefore, a ten equidistant point calibration curve was prepared in 0.9 % NaCl (w/v) for each analyte. The calibration curves ranged from 0.5 to 5 µg/L for DAI and GEN, from 0.2 to 2 µg/L for BPA, and from 0.1 to 1 µg/L for the remaining analytes. Precision and repeatability were revealed by the determination of intra- and inter-day relative standard deviation. By analysis of Q_low_ and Q_high_ ten times in one set of samples, precision in series was calculated. After the analysis of one Q_low_ and one Q_high_ sample on three different days, repeatability was determined. For the resulting coefficients of variation, a maximum tolerated value of 15 % was assumed. For the absolute and relative recoveries, four different urine samples with varying creatinine contents were investigated in different approaches. Series A was analyzed as blank sample. Series B was spiked with 10 µg/L of BP1, BP3, BHB, BPA, EHB, 4NP, MHB, PHB, TCPy, and TCS as well as 100 µg/L DAI and GEN before sample preparation and was treated according to the standard procedure. Series C was spiked accordingly after the extraction immediately before derivatization to erase analyte losses during sample preparation.

For the determination of relative recoveries, the analyte concentrations in the samples spiked before sample preparation (c_calculated_) were calculated using the applied external calibration. Afterwards, the resulting concentrations were divided through the known, actual spiked concentration (c_spiked_) and multiplied by 100:

$${Rec}_{ rel }\left(\%\right)= \frac{{c}_{ calculated }(\mu g/L)}{{c }_{spiked} (\mu g/L)} x 100$$

In contrast, the analyte concentrations of the samples spiked before sample preparation (series B, *c*_*B*_) and of the samples spiked after extraction directly before derivatization (series C, *c*_*C*_) were determined for the calculation of the absolute recoveries. The analyte concentrations in the urine blanks (series A, *c*_*A*_) were subtracted from both series. Afterwards, the ratio was calculated and multiplied by 100:

$${Rec}_{ abs }\left(\%\right)= \frac{{c}_{B} (\mu g/L)-{c}_{A} (\mu g/L)}{{c}_{C} (\mu g/L)-{c}_{A} (\mu g/L)} x 100$$

### Contamination control

In order to identify possible contaminations with ubiquitously free BPA, or parabens from cosmetics applied to human skin, a blank value was included in every measurement series. Thereby, this contamination control contained water instead of urine and underwent the whole sample preparation process. The analyte contents of this sample should be kept as low as possible and ideally be below the LOQ. Acquired analyte concentrations in the blanks were included in the calculation and subtracted from the urine concentrations.

### Application of the method to urine samples of environmentally exposed humans

After successful method development and validation, the analysis procedure was applied to human urine samples which were donated by members of laboratory staff to verify the suitability of the established method. During sampling, the subjects went about their normal daily routines in order to reflect their real-life exposures to the investigated analytes. All test persons gave their written informed consent to their participation.

## Results and discussion

### Calibration and control material

Usually, human urine is the matrix of choice for the preparation of calibration and control material to monitor the renal excretion of analytes in human biomonitoring. However, since the here investigated analytes of interest are used in various applications, such as cosmetics (MHB, EHB, PHB, BHB), nail polishes, sun protection products or perfumes (BP1, BP3), and biocides (TCS) as well as food (DAI, GEN), humans are exposed to these chemicals through their daily routines [8–13, 39–41]. Thus, background levels of the investigated analytes in collected pooled human urine samples are often observed which specifically concerns the phytoestrogens genistein and daidzein [23]. Given the ubiquitous nature of isoflavones in our diets, control subjects conform themselves to a restricted nutritional protocol or must be relied upon to be extremely careful in their intake of food items prior to providing biological fluids for analyses [42]. However, background levels in pooled human urine used as matrix for reference and control material leads to analytical challenges in measuring ultra-trace concentrations of the compounds of interest [37]. For this purpose, the preparation of calibration and control material using two surrogate matrices including 0.9% sodium chloride solution and artificial urine were tested as alternatives for pooled human urine [36, 37].

**Fig. 2:**
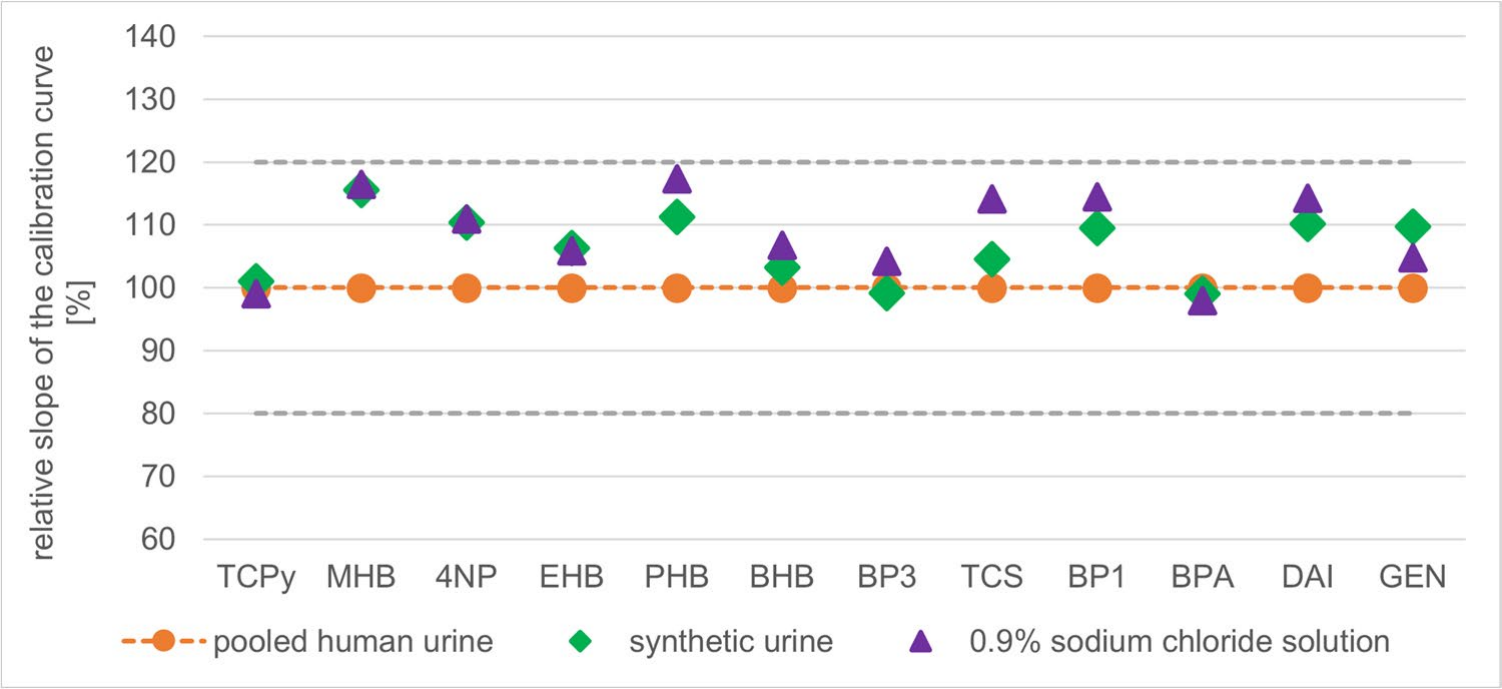
Relative slopes of the calibration curves in pooled human urine (orange), synthetic urine (green), and 0.9 % sodium chloride solution (violet) for all investigated analytes.

Comparison of the averaged calibration curves showed similar slopes for all matrices. Using the slope of the averaged calibration curve in pooled urine as a reference equal to 100%, acceptable values between 80 and 120% were obtained for all analytes in both alternative matrices which was in line with generally accepted criteria for matrix effects [43]. Figure 2 shows the relative slopes of the calibration curves in the compared matrices for all investigated substances. Thus, both synthetic urine and 0.9% sodium chloride solution are suitable and analyte-free surrogate matrices instead of pooled human urine. Finally, 0.9% NaCl was used as matrix for the preparation of the calibration and control material because of its effortless availability and lower costs.

### Sample preparation and derivatization (standard procedure)

The original sample preparation procedure developed by Schmidt [44] was adjusted to the newly added parameters BP1, BP3, MHB, EHB, PHB, BHB, and TCS. Again, precautions were taken including heat treatment of used glassware prior to analysis to eliminate sample contamination with environmentally occurring free BPA. Disposable nitrile gloves were worn during laboratory work to prevent possible contaminations with parabens from cosmetics applied to human skin. The hydrolysis step of the analysis procedure was adapted to guarantee an effective hydrolysis of both glucuronides and sulfates on the one hand but also to avoid ester cleavage of paraben structures on the other hand (see the “Optimization of the enzymatic hydrolysis” subsection). The subsequent solid phase extraction follows the originally developed method [44] as the extraction was proven applicable for the additional compounds. However, the eluates were concentrated to 100 µL, derivatized with MtBSTFA and directly measured via GC-MS/MS without further reduction of the volume to prevent evaporation losses of the more volatile silylated analytes.

### Exploration of the sample preparation and derivatization conditions

#### Applicability the derivatization procedure

For the derivatization with MtBSTFA, one specific peak was observed for every compound resulting from a *tert*-butyldimethylsilyl derivate of the considered analyte. For some analytes, the parent ions were detected, and others were measured as fragments of the original *tert*-butyldimethylsilyl derivates after the loss of methyl moieties (data not shown). As a result, MtBSTFA was considered as suitable for the derivatization of the recently added analytes BP1, BP3, MHB, EHB, PHB, BHB, and TCS. In total, the derivatization procedure was only slightly modified, as the SPE eluates were concentrated to 100 µL volume and directly derivatized without further concentration (see the “Sample preparation and derivatization (standard procedure)” subsection).

#### Optimization of the enzymatic hydrolysis

Enzymatic β‑glucuronidase/aryl sulfatase preparations from Helix Pomatia are known to cleave ester moieties of certain metabolites, such as phthalate and terephthalate metabolites [45–47]. Therefore, hydrolysis of phthalate and terephthalate conjugates was performed by using pure β-glucuronidase from *E. coli* K12 which was free of any aryl sulfatase/esterase activity. Accordingly, pure β-glucuronidase from *E. coli K12* was chosen to avoid the cleavage of the ester moieties of ethylhexyl salicylate (EHS) and its metabolites [48]. As only glucuronide conjugates were observed in human urine for phthalates and EHS metabolites, the lack of sulfatase activity had no incidence on the accuracy of the analysis [45, 48, 49]. In contrast, significant shares of sulfate conjugates were observed for the currently investigated analytes in previous metabolism studies [50, 51]. In general, the shares of parent parabens (free plus conjugated) excreted in urine decreased with increasing chain length of the alkyl moiety of the paraben, possibly due to different water solubility. Considerable sulfate levels were also observed in this study for the respective analytes (see Table 1). Thereby, the sulfate shares ranged between 0.3% for TCPy to 29.6% for NP. Among parabens, sulfate shares ranging from 15.5% for MHB to 6.9% for BHB were calculated. In contrast, Moos et al. [51] observed sulfate proportions of 63.4% and 13.5% for MHB and BHB, respectively. However, they calculated the total amount of free, glucuronide, and sulfate analyte after hydrolysis for 3.5 h at 37 °C by using β-glucuronidase/arylsulfatase HP2 from *Helix pomatia*. Thereby, they did not investigate the influence of possible ester cleavage reactions of parabens by lipases. If such analyte losses would have to be considered for these hydrolysis conditions, the sulfate shares would have been overestimated in the calculation, since the glucuronide components were determined without any possible lipase influence, in contrast to the total quantity. This could explain the discrepancies to the calculated sulfate proportions of the present method, for which only lipase-free enzymes were used.

**Table 1:** Sulfate proportions of the investigated analytes after hydrolytic cleavage in human urine.

Analyte	Sulfate proportion (%)
TCPy	0.3
4NP	29.6
MHB	15.5
EHB	12.0
PHB	13.9
BHB	6.9
BP1	9.6
BP3	13.2
TCS	13.3
BPA	5.7
DAI	2.3
GEN	5.1

**Fig. 3:**
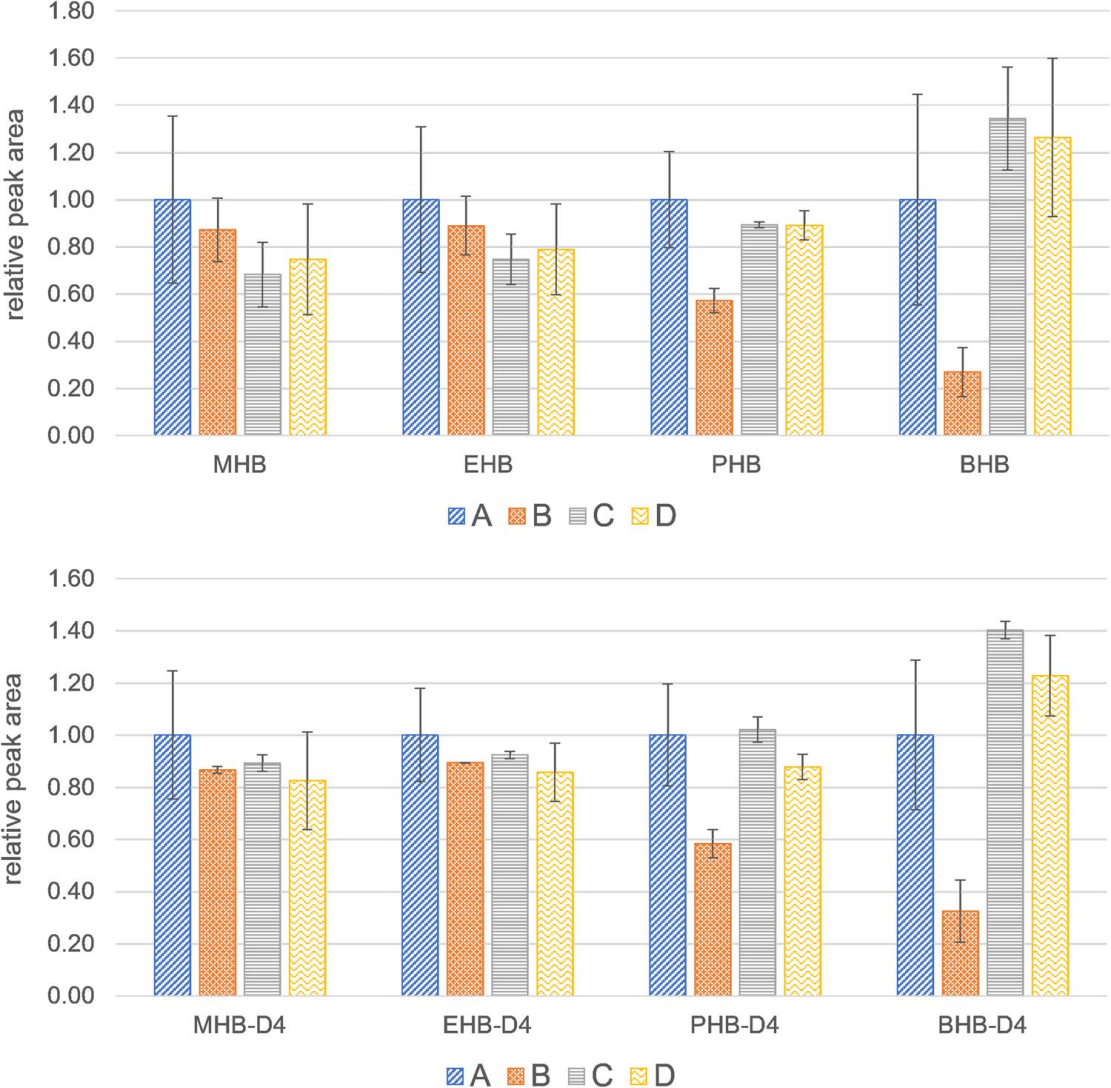
Relative peak areas of MHB, EHB, PHB, and BHB as well as their internal standards MHB-D4, EHB-D4, PHB-D4, and BHB-D4 for different hydrolysis procedures: (A) 10 µL β-glucuronidase/arylsulfatase from *Helix pomatia pH 5*, (B) β-glucuronidase Type H-1 from Helix pomatia pH 5, (C) 10 µL β-glucuronidase from *E. coli* K12, and (D) 10 µL β-glucuronidase from *E. coli K12* together with 10 µL sulfatase from *Aerobacter aerogenes Type VI pH 5*.

In the first approach, deconjugation with 10 µL β-glucuronidase/arylsulfatase from *Helix pomatia pH 5* (A) was compared to three further incubations, namely β-glucuronidase Type H-1 from *Helix pomatia pH 5* (B), 10 µL β-glucuronidase from *E. coli K12* pH 5 (C), and 10 µL β-glucuronidase from *E. coli K12* together with 10 µL sulfatase from *Aerobacter aerogenes Type VI pH 5* (D). Figure S1 shows the resulting response ratios for TCPy, NP, TCS, BPA, BP1, DAI, GEN, and BP3. Thereby, similar results were observed for the approaches (A), (B), and (D), while series (C) showed tendentially lower response ratios for some analytes, possibly caused by the lack of sulfatase activity. Nevertheless, the use of β-glucuronidase from *E. coli K12* combined with sulfatase from *Aerobacter aerogenes Type VI* delivered comparable results to the application of hydrolysis enzymes from *Helix pomatia.* However, the situation is different for the group of investigated benzoic acid esters. As both analytes and internal standards contain ester moieties, their responses were illustrated separately. Figure 3 shows the relative peak areas of MHB, EHB, PHB, and BHB as well as their internal standards MHB-D4, EHB-D4, PHB-D4, and BHB-D4. As the chain length of the alkyl radical increases from MHB to BHB, the differences between the various approaches become clearer: Thereby, especially PHB and BHB, together with their internal standards, show distinctly lower analyte responses for the series (A) and (B), where enzymes from *Helix pomatia* were used. This indicates cleavage of ester bonds of hydroxybenzoates by non-specific esterases, as previously observed for phthalates, therephthalates and EHS [45–48]. However, the use of β-glucuronidase from *E. coli K12*, especially in combination with sulfatase from *Aerobacter aerogenes Type VI*, offers a good alternative for enzymatic hydrolysis, which was further investigated in a second experiment.

**Fig. 4:**
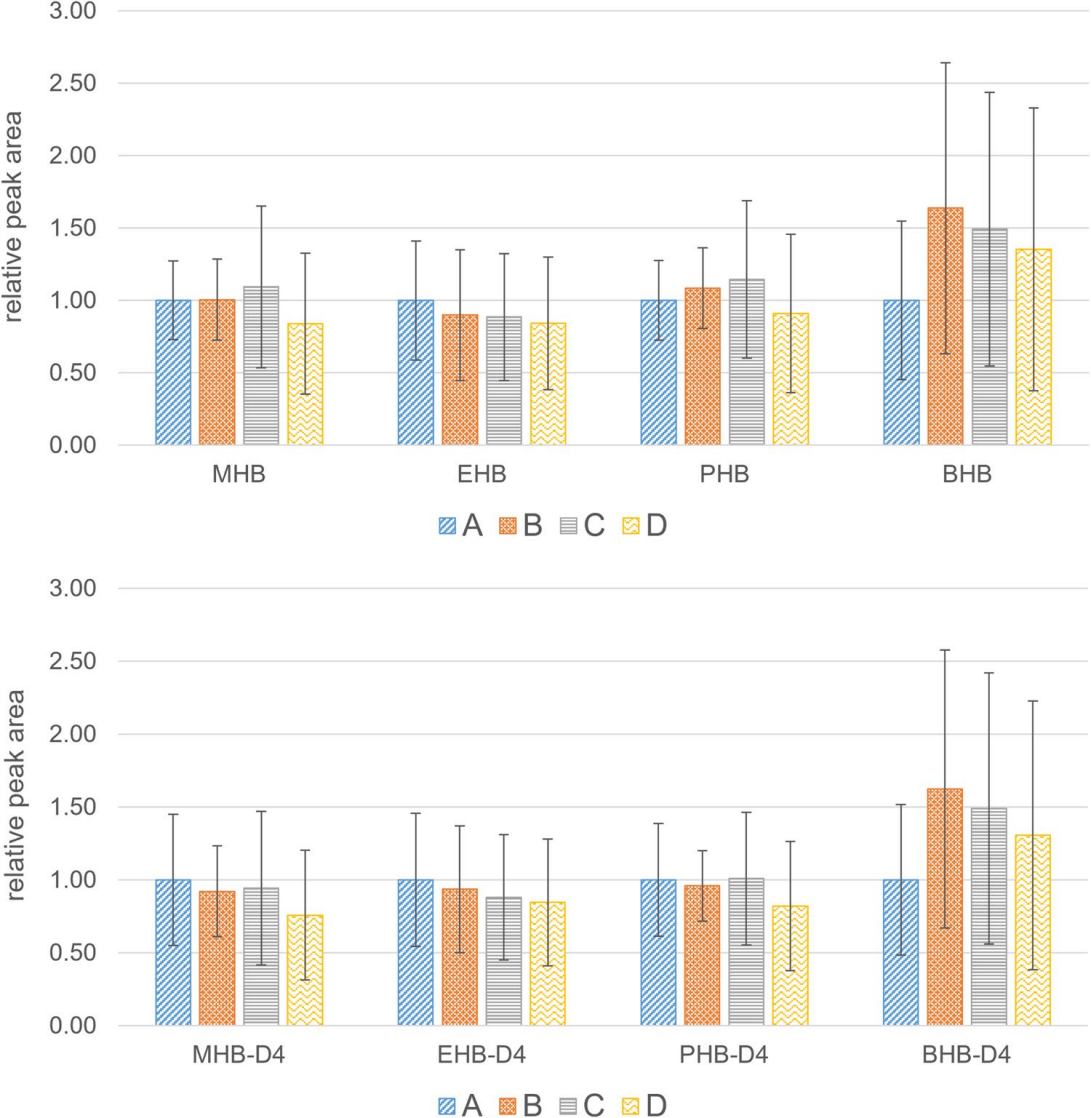
Relative peak areas of MHB, EHB, PHB and BHB as well as their internal standards MHB-D4, EHB-D4, PHB-D4, and BHB-D4 for different hydrolysis procedures: (A) 10 µL β-glucuronidase/arylsulfatase from *Helix pomatia pH 5*; 10 µL β-glucuronidase from *E. coli* K12 together with 10 µL sulfatase from *Aerobacter aerogenes* Type VI tested for pH 5 (B) and 6.5 (C). (D) 10 µL β-glucuronidase from *E. coli K 12* together with 50 µL sulfatase from *Aerobacter aerogenes* Type VI at pH 6.5

In the next step, another four different modifications were tested for the enzymatic hydrolysis. Again, deconjugation with 10 µL β-glucuronidase/arylsulfatase from *Helix pomatia pH 5* (A) was used as a reference. Furthermore, 10 µL β-glucuronidase from *E. coli K12* together with 10 µL sulfatase from *Aerobacter aerogenes Type VI* were tested for pH 5 (B) and 6.5 (C). Additionally, 10 µL β-glucuronidase from *E. coli K 12* together with 50 µL sulfatase from *Aerobacter aerogenes Type VI* were prepared at pH 6.5 (D). Figure S2 shows the resulting response ratios for TCPy, NP, TCS, BPA, BP1, DAI, GEN, and BP3. Again, similar responses were observed for these analytes in all four approaches A to D. Thus, the adjustment of the pH value to the optimum of 6.5 recommended for β-glucuronidase from *E. coli K12* by the manufacturer (Roche Diagnostics GmbH, Mannheim, Germany), did not negatively affect the determination of TCPy, NP, TCS, BPA, BP1, DAI, GEN, and BP3. Furthermore, the increase in enzyme volume from 10 to 50 µL sulfatase from *Aerobacter aerogenes Type VI* did not further enhance the analyte responses which indicates that a quantity of 10 µL is already sufficient for the cleavage of sulfate conjugates. A similar pattern was observed for the group of investigated benzoic acid esters (see Fig. 4). Thereby, the relative analyte responses for MHB, EHB, PHB, and BHB as well as their internal standards MHB-D4, EHB-D4, PHB-D4, and BHB-D4 were comparable for the approaches (B) to (D) and especially for BHB and BHB-D4 higher than approach (A). Thus, the adjustment to pH 6.5 had no adverse effects on the absolute peak areas of the hydroxy benzoates. Furthermore, an increased amount of sulfatase enzyme did not further enhance the yield of the enzymatic hydrolysis. Thus, the use of 10 µL β-glucuronidase from *E. coli K12* together with 10 µL sulfatase from *Aerobacter aerogenes Type VI* at pH 6.5 (C) was defined as the optimized standard procedure for the analysis of the investigated endocrine disruptive analytes.

### Gas chromatography-tandem mass spectrometry (GC-MS/MS)

**Fig. 5:**
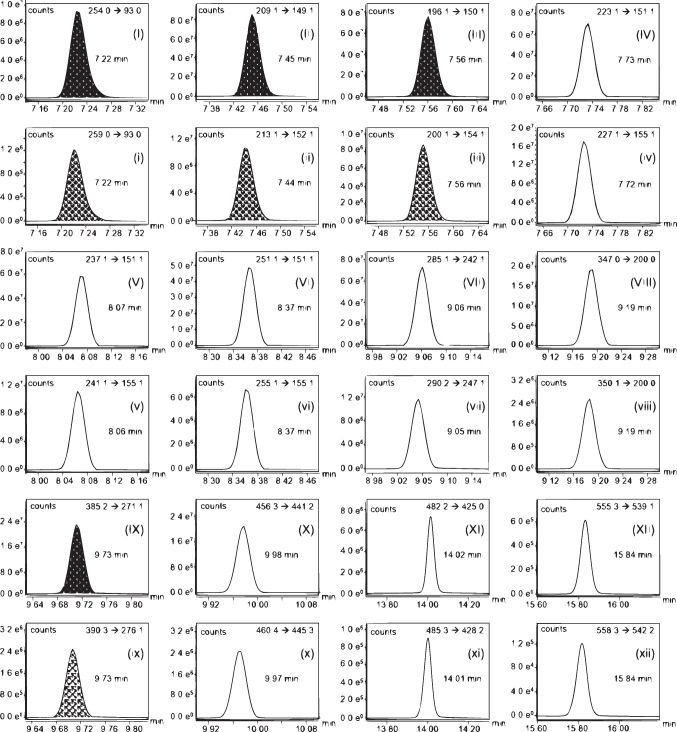
GC-AEI-MS/MS chromatogram of a calibration sample (50 µg/L BP1, BP3, BPA, MHB, EHB, PHB, BHB, NP, TCPy, and TCS as well as 150 µg/L GEN and DAI) shown as quantifier mass transitions of all investigated analytes and applied internal standards.

The derivatized analytes showed molecular weights ranging from 253 g/mol for NP to 612 g/mol for GEN (see Table S1). Thereby, the degree of derivatization was based on the number of available hydroxy groups. Thus, BHB, BP3, EHB, MHB, NP, PHB, and TCPy were silylated once, while BP1, BPA, and DAI were derivatized twice. The derivatized species of GEN contained three silyl groups. Consequently, it was a challenging task to determine a wide range of m/z ratios within one chromatographic run. At the same time, the four parabens had to be analyzed baseline separated, since these substances showed partly identical mass transitions due to their great structural similarity. As described by Schmidt [44], a steep temperature gradient was therefore elaborated to prevent peak broadening of the isoflavone signals and reduce analysis time. Finally, the investigated analytes showed baseline separation within an optimum runtime of 17.2 min. Figure 5 shows the quantifier mass transitions of a calibration sample of 50 µg/L BP1, BP3, BPA, MHB, EHB, PHB, BHB, NP, TCPy, and TCS as well as 150 µg/L GEN and DAI prepared and derivatized according to the standard procedure (see “Sample preparation (final procedure)” and “Derivatization process (final procedure)” sections) and measured via GC-AEI-MS/MS (see the “Gas chromatography-tandem mass spectrometry (GC-MS/MS)” section). High signal intensities of the investigated compounds were required to receive low limits of detection and quantification. Thus, the advanced electron ionization source enabled an improvement of sensitivity compared to a conventional electron ionization source [52]. The most common fragmentation patterns of the trimethylsilyl derivates were losses of methyl groups during the ionization process or the induced fragmentations in the collision cell. For NP, also the loss of a nitro moieties could be observed during the fragmentation process. Table S1 summarizes GC retention times and optimized MRM parameters for all analytes and internal standard substances.

### Reliability of the optimized method

Limits of detection were determined according to guideline DIN 32 645 and revealed values between 0.02 and 0.09 µg/L for BP1, BP3, BPA, BHB, EHB, MHB, 4NP, PHB, TCPy, and TCS (see Table 2). The corresponding limits of quantification varied between 0.08 and 0.29 µg/L. In contrast, higher limits of detection were calculated for DAI and GEN with values of 0.25 and 0.24 µg/L, respectively. Thus, limits of quantification were 0.83 and 0.79 µg/L for these two parameters. Nevertheless, the LOD and LOQ levels for GEN and DAI were sufficient, as much higher urinary concentrations were generally reported for both isoflavones [23]. In summary, excellent sensitivity of the method was proven for all investigated parameters.

**Table 2:** Validation data of the present method (estimated in 0.9% sodium chloride solution).

Analyte	LOD [µg/l]	LOQ [µg/l]	Precision (***n*** = 10)		Repeatability (***n*** = 10)		Relative recovery (***n***=4) [%]	Absolute recovery (***n***=4) [%]
			Q _low_ [%]	Q _high_ [%**]**	Q _low_ [%]	Q _high_ [%]		
TCPy	0.04	0.14	2.7	2.8	3.6	4.9	104.8 ± 0.6	93.2 ± 5.1
4NP	0.03	0.08	1.8	1.7	5.4	0.4	90.1 ± 7.9	91.5 ± 6.8
MHB	0.02	0.08	2.2	1.5	1.1	4.7	91.6 ± 5.5	81.7 ± 9.1
EHB	0.03	0.11	3.1	1.3	0.4	2.7	92.5 ± 5.8	99.9 ± 11.8
PHB	0.06	0.19	2.8	1.6	3.3	3.0	83.3 ± 7.5	94.3 ± 10.2
BHB	0.03	0.12	3.7	2.9	6.0	5.1	90.0 ± 12.4	95.2 ± 5.4
BP1	0.04	0.14	4.5	2.9	1.7	9.8	87.3 ± 4.1	73.5 ± 6.2
BP3	0.04	0.13	5.3	2.1	1.8	4.2	101.2 ± 11.0	83.6 ± 2.9
TCS	0.09	0.29	3.9	3.8	6.0	7.0	102.3 ± 10.6	73.5 ± 15.2
BPA	0.07	0.24	5.0	1.7	3.4	0.9	87.3 ± 4.8	89.8 ± 6.5
DAI	0.25	0.83	3.9	5.6	11.4	5.4	86.8 ± 8.5	88.0 ± 12.7
GEN	0.24	0.79	7.9	4.2	10.6	5.1	93.7 ± 14.0	91.6 ± 9.2

The variation coefficients for precision in series ranged from 1.8 to 7.9 % for Q_low_ and from 1.3 to 5.6 % for Q_high_, respectively. For the interday precision on three different days (*n* = 1), coefficients of variation between 0.4 and 11.4 % for Q_low_ and between 0.4 and 9.8 % for Q_high_ were calculated. Thus, very good intra- and inter-day precision of the procedure was proven.

Calculation of the relative recoveries revealed values between 83 and 105 % for all analytes. Thus, the relative recovery rates were in an acceptable range close to 100 %, proving good accuracy of the method. In contrast, absolute recoveries ranged from 73 to 100 %. Thereby, the lowest values were calculated for TCS and BP1. Still, the losses during sample preparation were acceptable as the relative recovery rates were close to 100 % for all analytes by use of structural-identical isotope-labeled internal standards. Additionally, good sensitivity of the analytical procedure was already proven through low LOD and LOQ values. The validation data of the optimized method is summarized in Table 2.

### Application of the method to urine samples of environmentally exposed humans

**Fig. 6:**
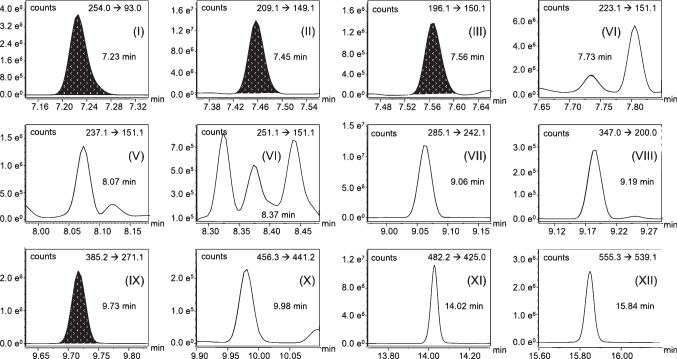
GC-AEI-MS/MS chromatogram of a representative spot urine sample. Sample preparation and analysis according to the final SOP. Detected metabolites shown as quantifier mass transitions.

The analysis of urine samples of environmentally exposed humans revealed different amounts of the considered substances, which is exemplary illustrated in Fig. 6. This chromatogram shows the quantifier mass transitions of the GC-AEI-MS/MS analysis for the detected analytes and their respective internal standards in a representative sample. In this urine specimen 2.6 µg/L TCPy, 1.2 µg/L NP, 7.7 µg/L BP3, 4.3 µg/L BP1, 1.3 µg/L BPA, and 1.2 µg/L TCS were detected. For the parabens, 20.4 µg/L MHB, 1.9 µg/L EHB, 1.3 µg/L PHB, and 0.6 µg/L BHB were calculated. Additionally, the isoflavones DAI and GEN were present in higher concentrations of 85.8 and 130.3 µg/L, respectively. For comparison, available biological assessment values of the investigated analytes were listed in Table 3. Therefore, the suitability of the developed method could not only be proven for pooled urine (see Table 2) but also for spot urine samples. The method can further be applied to monitor both occupational and environmentally exposed humans as the analytical procedure enables the detection and quantification with a broad calibration range up to 50 µg/L for BP1, BP3, BPA, BHB, EHB, MHB, 4NP, PHB, TCPy, and TCS as well as 150 µg/L for DAI and GEN for higher exposed specimen. Additionally, low limits of detection and quantification enable the ultra-trace analysis of the investigated possible endocrine disruptive compounds.

**Table 3:** Available biological assessment values of the investigated analytes

Substance	Biological assessment value	Value in urine
TCS [53]	HBM I	2 mg/L (children) 3 mg/L (adults)
MHB [53]	RV95	400 µg/L (women) 240 µg/L (men)
EHB [53]	RV95	50 µg/L (women) 25 µg/L (men)
PHB [53]	RV95	100 µg/L (women) 50 µg/L (men)
BHB [53]	RV95	20 µg/L (women) 10 µg/L (men)
BPA [54]	Health-based guidance value	100 µg/L (children) 200 µg/L (adults)
BP1 [55] TCPy [56]	LOAEL ADI	6 mg/mg bw/ day 0.06 mg/kg bw/day

### Comparison to other published methods

Table S2 compares the parameter spectrum and the sensitivity of the present method with other published procedures. Thereby, lower or at least comparable limits of detection and quantification were observed for the present method regarding the investigated analytes.

The present method enables the simultaneous determination of 12 possible endocrine agents, namely BPA, BP1, BP3, MHB, EHB, PHB, BHB, NP, TCPy, TCS, GEN, and DAI. Schmidt et al [44] previous published a multicomponent method for the simultaneous analysis of BPA, TCPy, GEN, and DAI. Thereby, β-glucuronidase/arylsulfatase from *Helix pomatia* was applied for enzymatic hydrolysis at 37°C over night. Afterwards, the analytes were extracted by solid phase extraction, derivatized by silylation with MtBSTFA and measured via GC-MS/MS. However, the use of a crude solution of β-glucuronidase/arylsulfatase from *Helix pomatia* may lead to sample contamination with GEN and DAI, since the gastric juice of these snails can contain isoflavones [35]. By use of β-glucuronidase from *E. coli* K12, especially in combination with sulfatase from *Aerobacter aerogenes* Type VI in the present method, no contaminants of isoflavones were introduced into the investigated samples. Moors et al. [57] investigated BPA, GEN, and DAI in urine after SPE using GC-MS analysis. Thereby, β-glucuronidase and sulfatase from *E. coli* were added for enzymatic hydrolysis at 37 °C over night. Consequently, they only used lipase-free and contaminant-free enzymes. Nevertheless, the presented method encompasses a much broader analytical spectrum than BPA, GEN, and DAI. Moos et al. [51, 58] used β-glucuronidase/arylsulfatase Type HP2 from *Helix pomatia* and an incubation duration of 3.5 h at 37 °C for hydrolysis prior to the analysis of MHB and BHB. Likewise, Dewalque et al. [59] performed hydrolysis of paraben conjugates (MHB, EHB, PHB, and BHB) by use of β-glucuronidase/arylsulfatase Type HP2 from *Helix pomatia* but with an overnight incubation at 37 °C. Thus, lipase activity was not excluded in both cases completely. However, shortening the hydrolysis time to 3.5 h may result in the cleavage of glucuronides and sulfates occurring preferentially first, while ester cleavage might be less apparently within this smaller period. Nevertheless, the analytical losses of the parabens during enzymatic hydrolysis were not investigated in both studies. Ye et al. [60] determined MHB, EHB, PHB, and BHB in urine after hydrolysis at 30 °C for 4 h with β-glucuronidase/sulfatase H1 from *Helix pomatia*. In the present study, the hydrolysis step was investigated using this enzyme for an overnight incubation (see the “Optimization of the enzymatic hydrolysis” subsection). Thereby, ester cleavage of parabens was observed for BHB in particular. However, the shorter hydrolysis time of 4 h may also result in the preferred deconjugation of glucuronides and sulfates as discussed for Dewalque et al. [59]. Nevertheless, the stability of the parabens in the hydrolysis step of Ye et al. [60] was not investigated more closely. Tkalec et al. [61] applied β-glucuronidase/arylsulfatase Type H2 from *Helix pomatia* and subsequently incubated the samples at 37 °C for 18 h for the analysis of BPA, MHB, EHB, PHB, BHB, and TCS*.* Thereby, the conditions of the enzymatic hydrolysis were verified regarding their applicability for parabens. The resulting deconjugation stability confirmed the absence of nonspecific enzymatic events in the hydrolysis step. Thus, it appears to be possible to use certain enzymes of *Helix pomatia* for the hydrolysis of paraben conjugates, but this requires an extremely deliberate and accurate execution of the method to ensure its robustness. In contrast, the advantage of the present method is that the enzymes used are absolutely lipase-free and the method therefore offers reliable reproducibility by preventing for analyte losses and therefore underestimating of the investigated compounds

Vela-Soria et al. developed two analysis procedures for the determination of MHB, EHB, PHB, BHB, BP1, BP3, and BPA via GC-MS/MS [62] and LC-MS/MS [63]. For both procedures, comparable low limits of detection were determined. Here, the GC-MS/MS method revealed tendentially higher sensitivity in comparison to the LC-MS/MS procedure. Moreover, gas chromatography provides generally higher chromatographic resolution and sharper peaks than liquid chromatography.

Some of the investigated analytes are known to ubiquitously occur in significant concentrations in human spot urine samples or pooled human urine. Thus, synthetic urine was already an established surrogate matrix for the analysis of BPA, TCS, and parabens as well as GEN and DAI [36, 59–64]. By using this analyte-free surrogate matrix, the quality of calibration and control material were positively affected, and especially the limits of detection and quantification were improved. However, a proof of the applicability of this approach is almost missing in the publications [36, 51, 58–64]. For the present method, both synthetic urine as well as 0.9 % sodium chloride solution were investigated as surrogate matrices. As similar slopes for the calibration curves were observed for the investigated analytes in all matrices, 0.9 % NaCl was used for calibration and control material as well as the determination of limits of detection and quantification (see the “Calibration and control material” subsection).

## Conclusion

The present method enables the simultaneous determination of 12 prominent endocrine agents, namely BPA, BP1, BP3, MHB, EHB, PHB, BHB, NP, TCPy, TCS, GEN, and DAI. Optimization of the enzymatic hydrolysis and the use of β-glucuronidase from *E. coli K12* as well as sulfatase from *Aerobacter aerogenes* enables the analysis of the total amounts of free compounds and their conjugates but also ensures the acquisition of intact analytes without cleavage of ester bonds among parabens. By using 0.9 % sodium chloride solution as matrix for calibration and control material, background levels of the analytes in the calibration and control material were avoided. The subsequent validation demonstrated a high reproducibility, accuracy, and sensitivity of the present method, which may be particularly derived from the use of structural-identical isotope-labeled internal standards for each parameter. Compared with previous published analytical procedures for the determination of the phenolic substances, the present method enables the simultaneous determination of a broad spectrum of biomarkers. Thereby, competitive or improved analytical sensitivity was ensured by use of GC-MS/MS with advanced electron ionization. Altogether, the procedure can be applied for exploring the exposome to these prominent endocrine disruptors in the general population.

## Supplementary information

Below is the link to the electronic supplementary material.

Supplementary file1 (PDF 386 KB)
