# Supplementary material for: Comprehensive monitoring of a special mixture of prominent endocrine disrupting chemicals in human urine using a carefully adjusted hydrolysis of conjugates
Source: Anal Bioanal Chem. 2022 Nov 26;415(4):555–70. doi: 10.1007/s00216-022-04438-0 (PMC9839815; doi:10.1007/s00216-022-04438-0)
Supplement: Supplementary file 1 — Supplementary file1 (PDF 386 KB) [file 216_2022_4438_MOESM1_ESM.pdf]

Supplemental material

Table S1 Retention times and MRM specific parameters of the analytes and internal standards.

analyte	t_R [min]	quantifier ion [m/z]			qualifier ion 1 [m/z]			qualifier ion 2 [m/z]		
		precursor ion (Q1)	product ion (Q3)	CE [V]	precursor ion (Q1)	product ion (Q3)	CE [V]	precursor ion (Q1)	product ion (Q3)	CE [V]
TCPy	7.22	254	93	15	256	93	15	254	219	10
TCPy-13C3	7.22	259	93	15	259	95	15	259	224	10
MHB	7.45	209	149	10	209	135	10	209	177	5
MHB-D4	7.45	213	152	10	213	139	10	213	153	10
NP-D4	7.56	200	154	10	200	139	20	200	153	15
NP	7.56	196	150	10	196	135	20	196	149	15
EHB	7.73	223	151	10	223	135	10	223	163	5
EHB-D4	7.72	227	155	10	227	139	10	227	167	5
PHB	8.07	237	151	5	237	195	5	294	237	5
PHB-D4	8.06	241	155	5	241	199	5	298	241	5
BHB	8.37	251	151	5	251	195	5	308	251	5
BHB-D4	8.37	255	155	5	255	199	5	312	255	5
BP3-D5	9.05	290	247	20	290	246	30	290	217	40
BP3	9.06	285	242	15	285	241	30	285	212	40
TCS-D3	9.19	350	200	15	350	202	15	350	185	35
TCS	9.19	347	200	15	345	200	15	347	185	35
BP1	9.73	385	271	20	385	355	15	385	369	20
BP1-D5	9.73	390	276	20	390	360	15	390	374	20
BPA-D4	9.97	460	445	10	460	209	20	445	235	20
BPA	9.98	456	441	10	456	207	20	441	233	20
DAI-D3	14.01	485	428	15	428	400	10	428	286	35
DAI	14.02	482	425	10	425	397	15	425	283	30
GEN-D4	15.84	558	542	35	558	486	35	559	543	35
GEN	15.84	555	539	35	555	483	35	556	540	35

Supplemental material

Optimization of the enzymatic hydrolysis

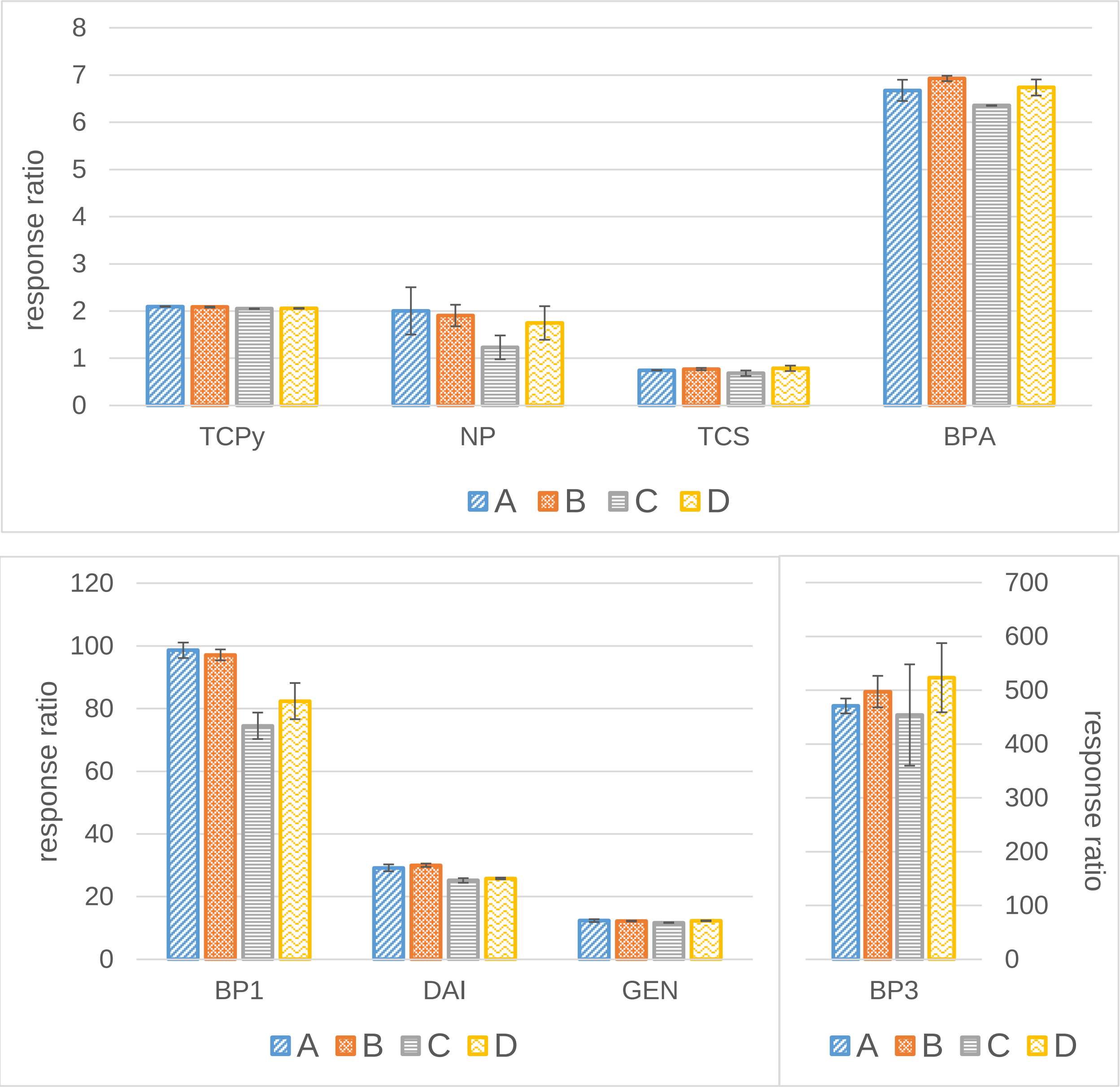

Figure S 1 Response ratios of the analyte peak areas to their respective internal standards for TCPy, NP, TCS, BPA, BP1, DAI, GEN and BP3 for four different hydrolysis approaches: (A) 10 µL β-glucuronidase/arylsulfatase from *Helix pomatia*, (B) β-glucuronidase Type H-1 from *Helix pomatia*, (C) 10 µL β-glucuronidase from *E. coli K12*, (D) 10 µL β-glucuronidase from *E. coli K12* plus 10 µL sulfatase from *Aerobacter aerogenes Type VI.*

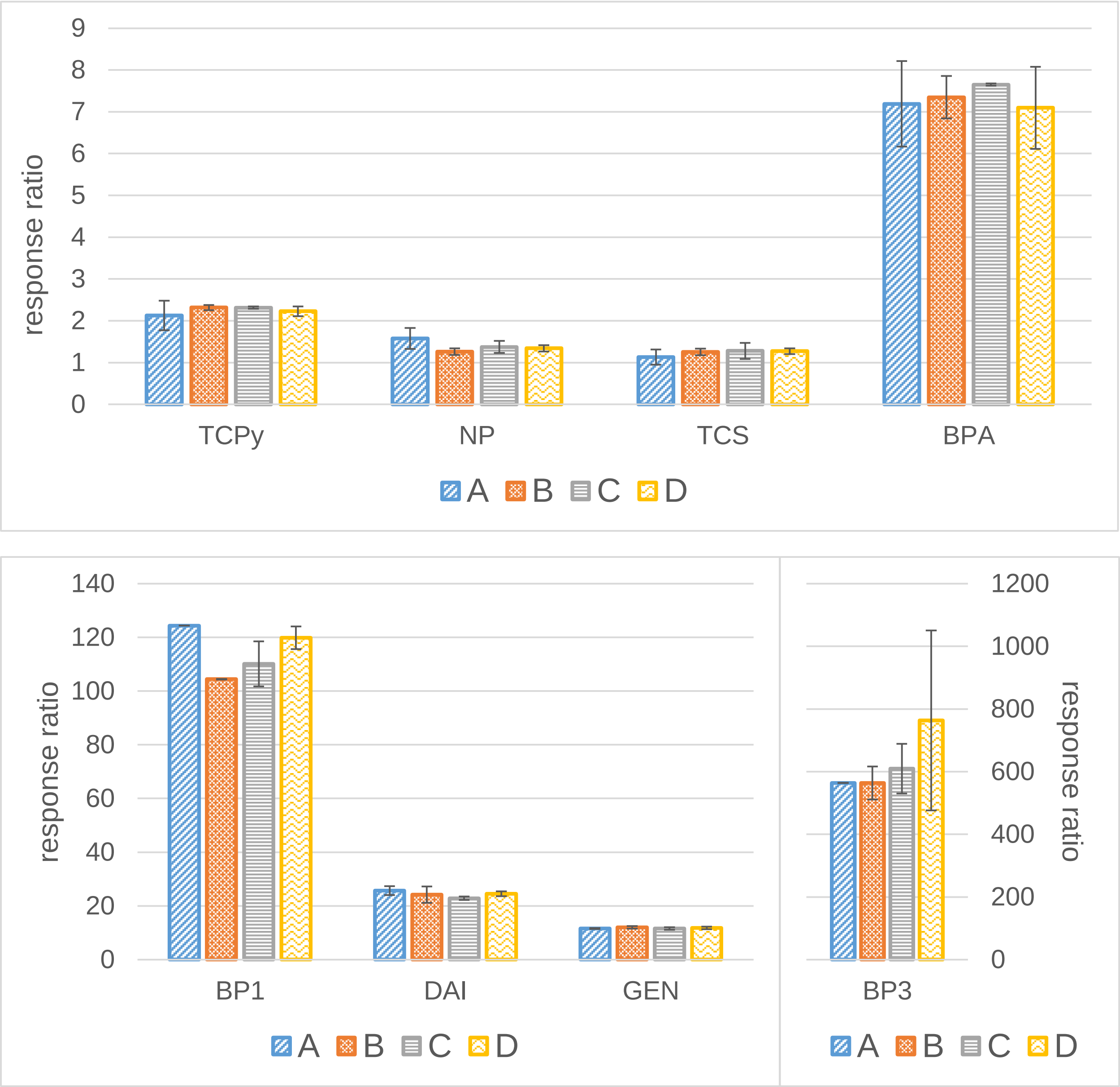

Figure S 2 Response ratios of the analyte peak areas to their respective internal standards for TCPy, NP, TCS, BPA, BP1, DAI, GEN and BP3 for four different hydrolysis approaches: (A) 10 µL β-glucuronidase/arylsulfatase from *Helix pomatia*, (B) 10 µL β-glucuronidase from *E.coli K12* together with 10 µL sulfatase from *Aerobacter aerogenes Type VI*, pH 5, (C) 10 µL β-glucuronidase from *E. coli K12* together with 10 µL sulfatase from *Aerobacter aerogenes* Type VI, pH 6.5, (D) 10 µL β-glucuronidase from *E.coli K 12* together with 50 µL sulfatase from *Aerobacter aerogenes* Type VI, pH 6.5.

Supplemental material

Table S2 Comparison of the parameter spectrum and the sensitivity of the present method with other published procedures.

**Analyte**	**Procedure, published by**											
LOD (LOQ) [µg/l]	Present method	Schmidt et al. 2013 [44]	Moors et al. 2007 [57]	Moos et al. 2014 [58]	Moos et al. 2016 [51]	Lee et al. 2013 [36]	Frederiksen et al. 2013 [64]	Tkalec et al. 2021 [61]	Vela-Soria et al 2014. [62]	Vela-Soria et al. 2014 [63]	Ye et al 2006 [60]	Dewalque et al. 2014 [59]
TCPy	0.04 (0.14)	0.2 (0.7)										
4NP	0.03 (0.08)											
MHB	0.02 (0.08)			0.5	0.25	0.30 (1.0)		(0.50)	0.06 (0.2)	0.1 (0.4)	0.13	0.16
EHB	0.03 (0.11)			0.5		0.18 (0.5)		(1.00)	0.08 (0.3)	0.1 (0.3)	0.10	0.09
PHB	0.06 (0.19)			0.5		0.08 (0.2)		(0.25)	0.08 (0.3)	0.1 (0.3)	0.18	0.11
BHB	0.03 (0.12)			0.5	0.25	0.16 (0.5)		(0.50)	0.1 (0.4)	0.1 (0.3)	0.10	0.3
BP1	0.04 (0.14)			0.5					0.1 (0.4)	0.2 (0.5)		
BP3	0.04 (0.13)			2.0			0.02		0.06 (0.2)	0.1 (0.5)		0.20
TCS	0.09 (0.29)			1.0			0.06	(0.25)				
BPA	0.07 (0.24)	0.1 (0.3)	3 (7)	0.5			0.12	(0.25)	0.2 (0.5)	0.2 (0.6)		
DAI	0.25 (0.83)	0.3 (1.1)	4 (9)									
GEN	0.24 (0.79)	0.6 (2.0)	5 (18)									

Supplemental material

LOD (LOQ) calculated	a	a	a	b	b	c	d	e	c	c	c	f
analytical technique	GC-MS/MS	GC-MS/MS	GC-MS	LC-MS/MS	LC-MS/MS	LC-MS/MS	LC-MS/MS	GC-MS/MS	GC-MS/MS	LC-MS/MS	LC-MS/MS	LC-MS/MS
calibration and control matrix	synthetic urine	pooled human urine	pooled human urine	Water (calibration standards) and human urine (quality controls)	Water (calibration standards) and human urine (quality controls)	synthetic urine	synthetic urine	synthetic urine	synthetic urine	synthetic urine	synthetic urine	synthetic urine

a LOD and LOQ determined according to DIN 32645 calibration function in urine; *b* S/N = 9; *c* LOD = 3 S_0, LOQ = 10 S_0; *d* LOD = 3 S_0; *e* LOQ = K1 with accuracy error of ≤ 20%; *f* LOD = LLOQ/3.3
